# WFS1 protein expression correlates with clinical progression of optic atrophy in patients with Wolfram syndrome

**DOI:** 10.1136/jmedgenet-2020-107257

**Published:** 2021-05-18

**Authors:** Kun Hu, Malgorzata Zatyka, Dewi Astuti, Nicola Beer, Renuka P Dias, Archana Kulkarni, John Ainsworth, Benjamin Wright, Anna Majander, Patrick Yu-Wai-Man, Denise Williams, Timothy Barrett

**Affiliations:** 1 Institute of Cancer and Genomic Sciences, University of Birmingham College of Medical and Dental Sciences, Birmingham, UK; 2 Oxford Centre for Diabetes, Endocrinology and Metabolism, Oxford University, Oxford, Oxfordshire, UK; 3 Institute of Metabolism and Systems Research, University of Birmingham College of Medical and Dental Sciences, Birmingham, UK; 4 Department of Ophthalmology, Birmingham Women's and Children's NHS Foundation Trust, Birmingham, UK; 5 Department of Neurology, University Hospitals Birmingham NHS Foundation Trust, Birmingham, UK; 6 Department of Ophthalmology, Helsinki University Hospital, University of Helsinki Faculty of Medicine, Helsinki, Uusimaa, Finland; 7 National Institute for Health Research Biomedical Research Centre at Moorfields Eye Hospital NHS Foundation Trust and UCL Institute of Ophthalmology, London, Greater London, UK; 8 Cambridge Centre for Brain Repair, University of Cambridge, Cambridge, Cambridgeshire, UK; 9 Department of Clinical Genetics, Birmingham Women's and Children's NHS Foundation Trust, Birmingham, Birmingham, UK; 10 Department of Endocrinology, Birmingham Women's and Children's NHS Foundation Trust, Birmingham, UK

**Keywords:** diabetes mellitus, genetics, medical, neurodegenerative diseases

## Abstract

**Background:**

Wolfram syndrome (WFS) is a rare disorder characterised by childhood-onset diabetes mellitus and progressive optic atrophy. Most patients have variants in the *WFS1* gene. We undertook functional studies of *WFS1* variants and correlated these with WFS1 protein expression and phenotype.

**Methods:**

9 patients with a clinical diagnosis of WFS were studied with quantitative PCR for markers of endoplasmic reticulum (ER) stress and immunoblotting of fibroblast protein extracts for WFS1 protein expression. Luciferase reporter assay was used to assess ATF-6 dependent unfolded protein response (UPR) activation.

**Results:**

6 patients with compound heterozygous nonsense mutations in *WFS1* had no detectable WFS1 protein expression; 3 patients with missense variants had 4%, 45% and 48% WFS1 protein expression. One of these also had an *OPA1* mutation and was reclassified as autosomal dominant optic atrophy-plus syndrome. There were no correlations between ER stress marker mRNA and WFS1 protein expression. ERSE-luciferase reporter indicated activation of the ATF6 branch of UPR in two patients tested. Patients with partial WFS1 expression showed milder visual acuity impairment (asymptomatic or colour blind only), compared with those with absent expression (registered severe vision impaired) (p=0.04). These differences remained after adjusting for duration of optic atrophy.

**Conclusions:**

Patients with WFS who have partial WFS1 protein expression present with milder visual impairment. This suggests a protective effect of partial WFS1 protein expression on the severity and perhaps progression of vision impairment and that therapies to increase residual WFS1 protein expression may be beneficial.

## Introduction

Wolfram syndrome (WFS), also known by the acronym DIDMOAD (diabetes insipidus, diabetes mellitus, optic atrophy and deafness; MIM#222300), is a rare autosomal recessive disease characterised by childhood-onset diabetes mellitus (DM) and optic atrophy (OA) associated with neuropathic bladder and neurodegeneration.^1 2^ The estimated prevalence is 1 in 770 000 in the UK.^1^

WFS is one manifestation of *WFS1*-related disorders, caused by variants in the *WFS1* gene.^3^ Other manifestations include *WFS1*-related low-frequency sensorineural hearing loss (*WFS1-*related LFSNHL), characterised by congenital, non-syndromic, low-frequency sensorineural hearing loss, and WFS-like disease, characterised by sensorineural hearing loss, DM, psychiatric illness and variable OA, not limited to childhood presentation.^4^ Both WFS1-related LFSNHL and WFS-like disease are dominantly inherited.^4–6^

The WFS1 protein (MIM#606201) is located in the endoplasmic reticulum (ER) membrane.^3^ One of its functions relates to the unfolded protein response (UPR) pathways, where it is upregulated in response to ER stress.^7^ ER stress occurs when the cellular demand for protein production exceeds the protein folding capacity in the ER.^8^ WFS1 is a negative regulator of the UPR.^9^ It binds to the ER stress sensor, ATF6, leading to its proteasomal degradation and preventing chronic activation of the UPR and cell death.^9^

The *WFS1* gene is located on the short arm of chromosome 4 at position 16.1 (4p16.1).^3^ There are currently 309 reported disease-causing *WFS1* variants.^5^ Most variants occur in exon 8 with the majority being nonsense, duplications or deletions resulting in early stop codons or additional translation of previously non-coding DNA.^5^

Previous studies have shown that patients with WFS who had an in silico predicted complete loss of WFS1 protein function had an earlier onset of WFS,^10^ DM^11^ and OA,^11^ compared with patients who had predicted partial loss of WFS1 function. Other studies showed patients with classic WFS had worse visual acuity and reduced retinal nerve thickness compared with patients with autosomal dominant WFS-like syndrome.^12^ WFS1 protein expression was measured in a single patient with neonatal diabetes insipidus (DI) and unilateral optic disc hypoplasia.^13^ This patient was found to have reduced WFS1 protein expression, but as this was due to a segmental paternal heterodisomy of chromosome 4, it is not clear whether other genetic defects were involved.

In the current study, we aimed to explore the functional consequences of known disease-associated variants as well as missense variants of unknown significance in patients referred to our service with a clinical diagnosis of WFS.

## Methods

### Patients

We defined WFS as the coexistence of childhood DM and OA (under 16 years).^1^ Patients were recruited from National Health Service England highly specialised national multidisciplinary service for WFS in Birmingham, UK, and participating in the EUROWABB registry (EU Rare Diseases Registry for Wolfram syndrome, Alström syndrome, Bardet-Biedl syndrome and other rare diabetes syndromes: http://euro-wabb.org/).^14^ Clinical symptoms were recorded, and severity was assessed using: glycated haemoglobin (HbA1C) for glycaemic control in DM; pure tone average calculated from 0.5, 1, 2 and 4 kHz and qualitative description of pure tone audiometry from 0.25 to 8 kHz for measurement of hearing loss^15^; and logMAR value for visual acuity. Data were collated using IBM SPSS Statistics 25, and groups were compared for statistical analysis with Student’s t-test for parametric data and the Mann-Whitney U test for non-parametric data.

### Gene variant analysis

Venous blood was collected for *WFS1* gene sequencing. This was initially carried out by Sanger sequencing with an ABI 3730 DNA sequencer, subsequently superseded by multiplex ligation-dependent probe amplification using ABI 3130 DNA sequencer and 3500 Genetic Analyzers. The following polymorphism prediction programmes were used for in silico analysis to predict the pathogenicity of *WFS1* missense variants: SIFT,^16^ PolyPhen-2,^17^ Mutation Taster^18^ and Provean.^19^

### Fibroblast culture

Primary fibroblasts were cultured at the Human Biomaterials Resource Centre University of Birmingham. Fibroblasts from healthy individuals were purchased from the European Collection of Cell Cultures: control 1 (C1) was from a 70-year-old white European man; control 2 (C2) and control 3 (C3) were from 46-year-old and 28-year-old white European women, respectively. The fibroblasts were cultured in Advanced DMEM medium (Life Technologies), supplemented with 10% fetal bovine serum Biosera), Penicillin-Streptomycin and Gluta-MAX (Life Technologies) and grown in 37°C/5% CO_2_ incubators. Cultures were grown to 80% confluency before use for the functional assays described below.

### Quantitative PCR for WFS1 mRNA and markers of ER stress (BiP, CHOP and sXBP1)

RNA from fibroblasts of patients and controls was prepared following the TRIzol protocol (Invitrogen). DNA was removed using DNA free kit (Ambion), and cDNA was prepared with High Capacity cDNA Reverse Transcription kit (Applied Biosystems). Quantitative PCR was performed using TaqMan Expression Assays (Applied Biosystems) for WFS1, BiP, CHOP and sXBP1. Results were calculated by delta-delta CT method and quantified as a percentage in relation to control levels. Experiments were repeated at least four times, and results were analysed by Student’s t-test.

### Immunoblotting for WFS1 protein expression

Fibroblasts from patients and controls were harvested in SDS lysis buffer (0.5M Tris pH7.0, 10% SDS, 25% glycerol). Ten micrograms of protein extract was run on SDS PAGE gels in Tris/glycine/SDS running buffer (Geneflow). Gel transfer to PVDF (Polyvinylidene difluoride) membrane was performed in Tris/glycine transfer buffer (Geneflow) at 90V for 1 hour. Incubation with primary anti-WFS1 antibody (Proteintech, rabbit polyclonal), at 1:1000 dilution in 5% milk/PBS-Tween, was performed overnight at 4°C. Secondary antirabbit antibody (Dako) was used at 1:20 000 for 1 hour at room temperature. Integrated optical density with Gene Tool software was used for quantification. WFS1 levels were quantified as a percentage in relation to control levels. Experiments were repeated four times, using two independently prepared sample extracts. Results were analysed by Student’s t-test.

### Luciferase reporter assay for ATF6-dependent UPR activation

#### Plasmids

‘E1T’ plasmid was an ER stress-response element (ERSE) reporter plasmid that encoded firefly luciferase downstream of a putative ERSE enhancer within the pGL3 Promoter plasmid (Promega).^20^ The ERSE sequence was subcloned in triplicate using BglII and SmaI restriction sites.

The internal control plasmid was Renilla-reporter plasmid pRL-SV40 (Promega), which was used to normalise for transfection efficiency.

#### Transfection and reporter assay

Fibroblasts from patients S02 and S10 were transfected with Fugene transfection reagent (Promega) and cotransfected with either: ‘E1T’ plasmid with the pRL-SV40 plasmid or pGL3 control plasmid with pRL-SV40 plasmid.

Forty-eight hours after transfection, the cells were harvested in Passive Lysis Buffer (Promega), and luciferase activity was measured using the Dual Luciferase Reporter Assay System (Promega). Bioluminescence was detected using a Centro LB 960 microplate luminometer (Berthold Technology). The results are presented as ‘relative luciferase activity’ (a ratio of the normalised value obtained for E1T plasmid to pGL3 control plasmid). This is a reflection of ER stress signalling and ATF6-dependent UPR. The mean values from at least four experiments were used and results analysed by Student’s t-test.

## Results

### Clinical presentation

**Figure 1:**
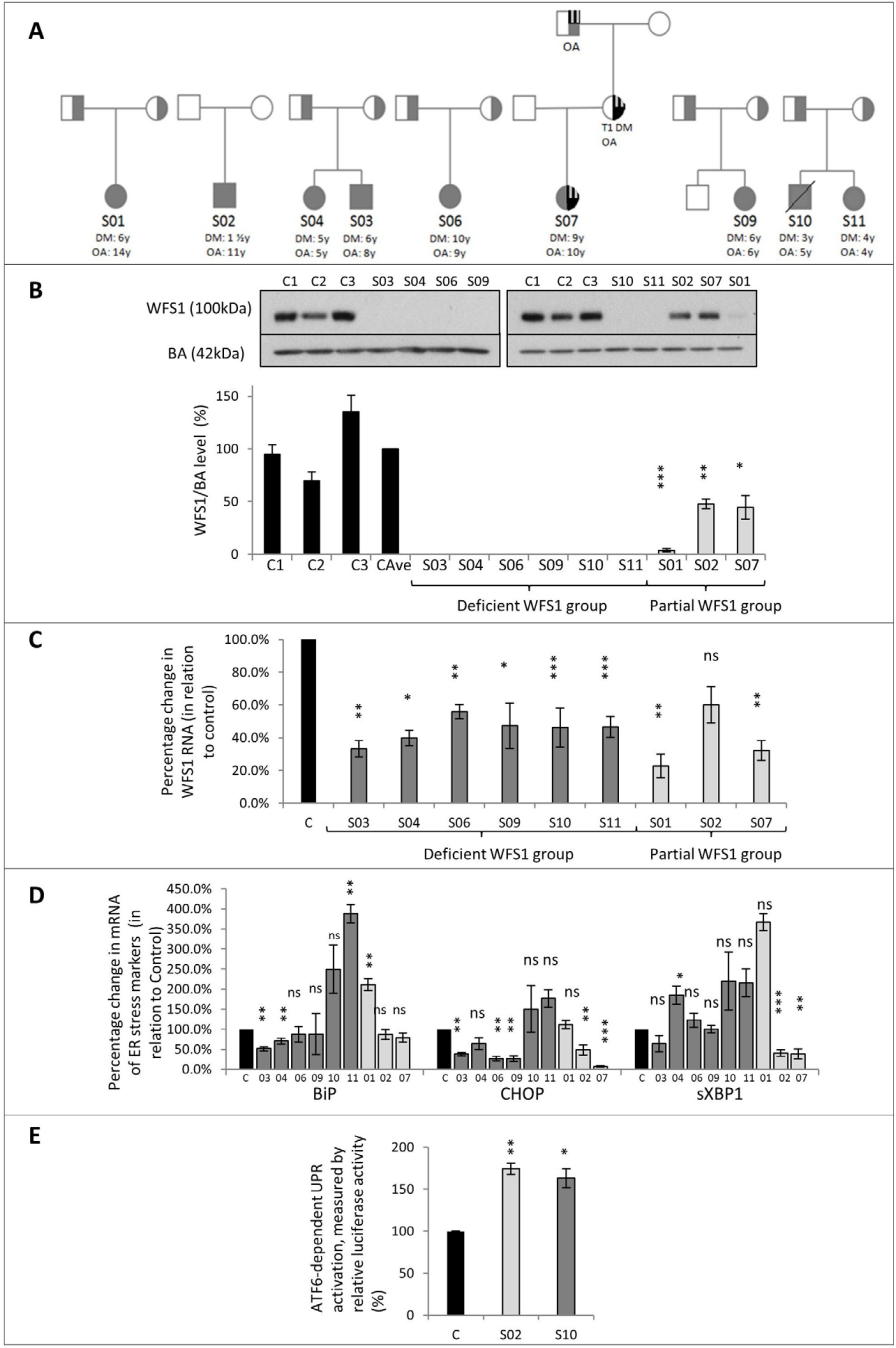
Family pedigrees and functional data.**(A)** Pedigrees of the 7 families reported in this study. All patients included in the study marked in grey. The age of onset of diabetes mellitus (DM) and optic atrophy (OA) stated. S02 had a de-novo mutation. For patient S07, OPA1 and WFS1 variants were found in all three generations in this family: S07’s maternal grandfather had isolated OA; S07’s mother had isolated OA and Type 1 DM. OA and DM in S07’s family represented by quarter stripes (OA) and quarter black (DM). All other patients inherited recessive alleles from each parent.**(B)** Immunoblotting images and corresponding bar chart with standard error bars showing levels of WFS1 protein. WFS1 and beta-actin (BA) protein levels measured in fibroblast from patients with WFS and healthy controls. C1, C2, C3 = healthy controls; CAve: average of controls. WFS1 levels for CAve=100%. WFS1 protein was undetectable in patients: S03, S04, S06, S09, S10 and S11. WFS1 protein was reduced in S01, S02 and S07 by 96.2%, 53.3%, and 55.4% respectively in comparison to CAve. Analysis by Student's T-test.**(C)** Bar chart with standard error bars showing quantitative PCR analysis of WFS1 mRNA, as percentage change when standardised with control. C=control. (n= 4) Analysis by Student's T-test.**(D)** Bar chart with standard error bars showing quantitative PCR analysis of ER stress marker mRNA: BiP, CHOP and sXBP1, as percentage change, when standardised with control (C). (n=4). Dark grey bars indicate patient in the deficient WFS1 protein group, and the light grey bar indicates the patient is in the partial WFS1 protein group. Analysis by Student's T-test.**(E)** Bar chart with standard error bars showing quantification of ATF6-dependent UPR activation by ERSE luciferase reporters, for SO2 and S10, as a percentage change compared from control (C). (n=4) Analysis by Student's T-test.ns: P >0.05; * P≤0.05; ** P ≤0.01; *** P≤0.001 compared with control samples

Nine patients with a clinical diagnosis of WFS from seven unrelated families were recruited. Patients S03 and S04 were siblings, as were patients S10 and S11 (figure 1A, table 1). There were six females and three males (age 17–32 years). The median age of onset of:DM was 6 years (range 3–10 years); OA was 6 years (4–14),;hearing loss was 8 years (birth–15); DI was 13 years (3–16) in five patients; and urinary dysfunction was 15.5 years (10–16) in six patients.

**Table 1:** Clinical features of all patients with WFS included in this study

Family	1	2	3	4		5	6	7	
Patient	S01	S02	S07	S03	S04	S06	S09	S10	S11
Sex	F	M	F	M	F	F	F	M	F
Consanguinity	No	No	No	No	No	No	No	No	No
Current age (years)	21.4	17.4	17.5	21.9	26.0	25.2	17.4	31 RIP	32.7
BMI (kg/m^2^)	24.9	22.5	26.8	19.3	21.2	34.8	21.8	N/A	N/A
DM (years)	6	1.5	9	6	5	10	6	3	4
Mean HbA1c (mmol/mol)	65.2	79.0	64.2	55.2	64.7	79.8	61.3	75.0	62.0
OA: age at diagnosis (years)	14	8	4	8	5	9	5	5	4
Current logMAR* value	0.4	0.2	0.3	1.6	2.2	1.8	1.7	2.9 (no light perception)	2.9 (no light perception)
Hearing loss onset (years)	14	1.5	0	8	4	12	13	9	6
Pure tone average (dB); (qualitative description of audiogram)	15 (0–10 dB from 0.25 to 2 kHz, 30 dB at 4 Hz, 55 dB at 8 kHz)	75 (70–80 dB from 0.25 kHz onwards)	112 (90 dB at 0.25 to 0.5 kHz, 120 dB from 1 kHz onwards)	65 (40–50 dB at 0.25 kHz, increasing dB from 0.25 kHz onwards, 90–100 dB at 8 kHz	85 (50 dB at 0.25 kHz, 80 dB from 0.5kHz to 2kHz, 100 dB from 3 kHz onwards)	10 (0–10 dB from 0.25 to 4 kHz, 55 dB at 8 kHz)	10 (0–10 dB from 0.25–4 kHz, 35 dB at 8 kHz)	70 (40 dB at 0.25–0.5 kHz, increasing dB from 0.5 kHz onwards, 100 dB at 8 kHz)	57 (40 dB at 0.25–1 kHz, 70 dB at 2 kHz, 80 dB from 4 kHz onwards)
DI onset (years)	13	None	None	None	16	None	15	3	6
Urinary dysfunction onset (years)	15	14	No	16	16	16	7	Neuropathic bladder	Neuropathic bladder
Degree of urinary dysfunction	Staccato void, megacystis (improving)	Staccato void, megacystis (improving)	None	Neurogenic bladder	Neurogenic bladder	Neurogenic bladder, recurrent UTIs	Neurogenic bladder	Self-catheterising	Self-catheterising
Neurological/psychiatric symptoms	Depression, night terrors	Headaches	None	Mild bilateral hand tremor, social anxiety and vivid dreams	Previous auditory/visual hallucinations, migraine-type headaches, sleep disturbance and anxiety	Bulbar palsy, depression, marked balance problem, previous self-harm, obsessive-compulsive features and headaches	Bulbar palsy, dyssynergic defecation and mood swings	Depression, restless legs, myoclonic jerks, ataxia and chronic fatigue syndrome	Chronic fatigue syndrome
MRI brain report	Atrophy of optic nerve, chiasm and tracts	Atrophy of optic nerve, chiasm and tracts	MRI not undertaken	Atrophy of optic nerve, chiasm and tracts	Atrophy of optic nerve, chiasm and tracts	Atrophy of optic nerve, chiasm and tracts	Atrophy of optic nerve, chiasm and tracts	MRI not undertaken	Atrophy of optic nerve, chiasm and tracts
Other	Underactive thyroid	Learning impairment	None	None	Gastritis, nausea and primary ovarian failure	Oropharyngeal dysphasia	Sleep apnoea, tracheostomy (grade 1 laryngeal cleft), previous nasogastric (NG) tube fed	N/A	Depression wheelchair-bound due to falls. On antireflux medicine

*LogMAR value (visual acuity logarithm of the minimum angle of resolution) is the magnification requirement; the higher the logMAR value, the worse the visual acuity (<1.0: mild to moderate visual impairment, 1.0–1.3: sight impaired (partial sighted), >1.3: severely sight impaired (blind).^40–42^

BMI, body mass index; DM, diabetes mellitus; HbA1c, glycated haemoglobin; OA, optic atrophy; WFS, Wolfram syndrome.

### Variant analysis

**Figure 2:**
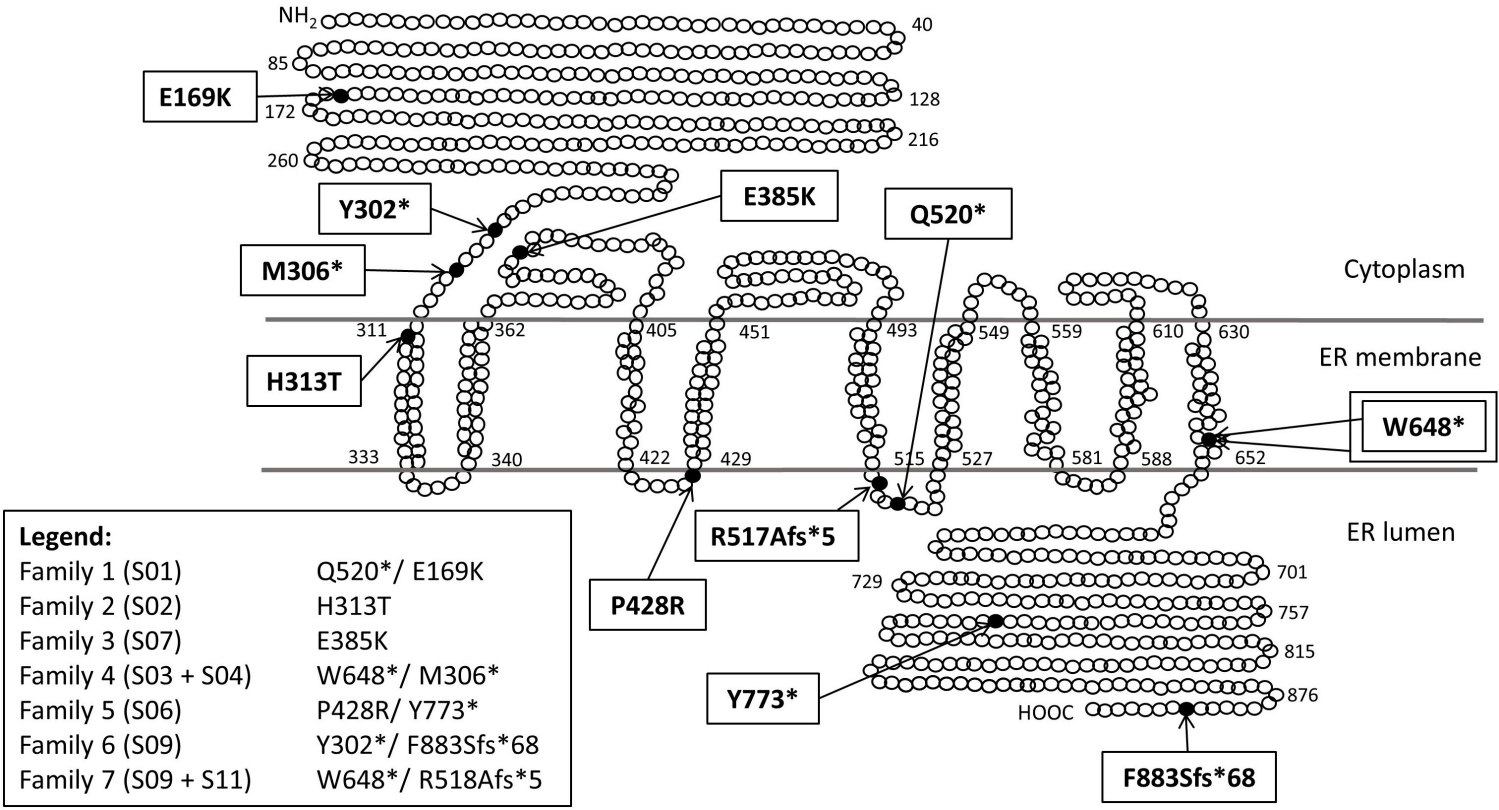
Schematic representation of WFS1 protein and variant locations.

The location of *WFS1* variants detected is shown in figure 2, and details of *WFS1* variant analysis are shown in table 2.

**Table 2:** WFS1 variants seen in patients with WFS reported in this study, in silico analysis for polymorphism prediction and experimentally measured WFS1 protein level

Family	Patient	Nucleotide change	Amino acid change	WFS1 protein location	Type of variant	Disease-associated polymorphism prediction analysis				WFS-1 protein
						SIFT	PolyPhen-2 HumVar	Mutation taster	Provean	
1	S01	c.505G>A	p.Glu169Lys	Cytosolic N-terminus	Missense	0.1 predict tolerated	0.972 probably damaging	Disease causing	−1.312 Neutral	3.8%
		c.1558C>T	p.Gln520X	Luminal loop III	Nonsense	N/A	N/A	N/A	N/A	
2	S02	c.937C>T	p.His313Tyr	Trans-membrane domain I	Missense	0.11 predict tolerated	0.628 possibly damaging	Disease causing	−0.651 Neutral	47.7%
		c.1709_14dupTGCCCC	Within minimal promotor region	Outside coding region (minimal promoter region)	Duplication	N/A	N/A	N/A	N/A	
3	S07	c.1153G>A	p.Glu385Lys	Cytosolic loop I	Missense	0.12 predicted tolerated	0.403 benign	Disease causing	−1.865 Neutral	44.6%
		Wild type	Wild type	N/A	Wild type	N/A	N/A	N/A	N/A	
		Duplication of exons 4–8 in OPA1	Disease-associated OPA1 variant	N/A	Duplication	N/A	N/A	N/A	N/A	
4	S03 + S04	c.911_914 dup TTGA	p.Met306X	Cytosolic N-terminus	Nonsense	N/A	N/A	N/A	N/A	0.0%
		c.1944G>A	p.Trp648X	Transmembrane domain IX	Nonsense	N/A	N/A	N/A	N/A	0.0%
5	S06	c.2319C>G	p.Tyr773X	C-terminal ER luminal domain	Nonsense	N/A	N/A	N/A	N/A	0.0%
		c.1283C>G	p.Pro428Arg	Luminal loop II	Missense	0 predict deleterious	0.995 probably damaging	Disease causing	−7.509 Deleterious	
6	S09	c.2648_2651delTCTT	p.Phe883SerfsX68	C-terminal ER luminal domain	Frameshift	N/A	N/A	N/A	N/A	0.0%
		c.906C>A	p.Tyr302X	Cytosolic N-terminus	Nonsense	N/A	N/A	N/A	N/A	
7	S10 + S11	c.1549delC	p.Arg517AlafsX5	Luminal loop III	Frameshift	N/A	N/A	N/A	N/A	0.0%
		c.1944G>A	p.Trp648X	Trans-membrane domain IX	Nonsense	N/A	N/A	N/A	N/A	0.0%

SIFT (0.0–0.05 considered deleterious; 0.05–1.0 predicted tolerated (benign)). Polyphen-2 (0.0–0.15 predicted benign; 0.15–1.0 possibly damaging; 0.85–1.0 more confidently predicted damaging). Provean (≤−2.5 ‘deleterious’; ≥−2.5 ‘neutral’. Polymorphism prediction software consulted in March 2019. Polymorphism prediction software consulted in March 2019.

Compound heterozygous *WFS1* mutations were found in seven patients. S03, S04, S09, S10 and S11 all had nonsense or frameshift variants, previously reported to be disease associated.^5^

S01 and S06 had nonsense and missense *WFS1* variants in trans. The missense variant in S01 (c.505G>A;p.Glu169Lys) has been previously reported^21 22^ and predicted to be damaging by PolyPhen-2 and Provean. The missense variant in S06 (p.Pro428Arg) has also been previously reported^5^ and predicted to be damaging in all prediction programmes.

Patient S02 had a missense variant (p.His313Tyr) that was ‘de novo’, that is, not present in the parents. The variant has been previously reported^6 23^ and shown to be disease associated and autosomal dominantly inherited.^24^ The other allele had a 6 bp duplication in a non-coding region, inherited from their asymptomatic mother.

Patient S07 had a previously reported missense variant (c.1153G>A; p.Glu385Lys)^5 25–27^ and a wild type WFS allele. This variant was only predicted to be disease-associated in *Mutation Taster* software. Due to a family history of OA in mother and maternal grandfather, a search was made for other genetic causes of OA. A disease-associated duplication (exon 4–8) in the *OPA1* gene was subsequently identified (figure 1A). On further investigation, S07 was noted to have positive antibodies to glutamic acid decarboxylase (GAD), associated with type 1 diabetes. Patient S07 was subsequently reclassified as autosomal dominant optic atrophy (DOA) with profound early-onset deafness and type 1 (autoimmune) diabetes.

### WFS1 mRNA levels

Real-time PCR results (figure 1C) showed a 39.8% (SE±0.12) to 77.3% (SE±0.15) reduction in *WFS1* mRNA expression in all patients with WFS compared with control samples.

### WFS1 protein levels

The three controls had variable WFS1 protein levels (figure 1B), so the mean value was used for comparisons (CAve).

No WFS1 protein was detected on immunoblotting of fibroblasts from S03, S04, S06, S09, S10 and S11. Detectable but reduced expression of WFS1 protein was observed in fibroblasts from S01 (3.8%; SE±1.4; p<0.001), S02 (47.7%; SE±4.7 p<0.02) and S07 (44.6%; SE±11.2 p<0.01), compared with CAve.

### ER stress levels

There was a large variation in the expression of ER stress markers in all patients with WFS (figure 1D). S04, S10 and S11 displayed increased expression of at least one marker, although these did not all reach statistical significance. In patients S02, S03, S06, S07 and S09, the markers were unchanged or decreased.

There were no correlations observed between the mRNA levels of the ER stress markers and WFS1 protein expression or severity of WFS phenotype.

ATF6-dependent UPR activation was measured with ERSE-luciferase reporter in fibroblasts of patients S02 and S10. In both, the luciferase levels were increased under steady-state conditions by 63% (SE±6.5; p=0.03) and 74% (SE±11.3; p=0.001), respectively (figure 1E). This indicates the activation of the ATF6 branch of UPR.^20^ Both patients had similar profiles of UPR activation despite having differing levels of WFS1 protein. In the patient with WFS1 protein expression, this may reflect the dominant-negative effect of the p.His313Tyr gene variant.

### Genotype–phenotype correlations

Patients who had no detectable WFS1 protein expression (the ‘deficient WFS1 protein’ group) all had *WFS1* variants that were either nonsense, frameshift or previously reported and had predicted disease-associated missense mutations on in-silico analysis. These six patients have the following median ages of onset: DM 5.5 years (range 3–10 years); OA 5.5 years (4–9); DI 10.5 years (3–16); hearing loss 8.5 years (4–13); urinary dysfunction 16 years (10–16). In terms of the disease severity, the median HbA1c was 63.4 mmol/mol (55.2–79.8), the median hearing loss range was 77.5 dB (35–100) and the median logMAR value for current visual acuity was 2.0 (1.7–2.9).

The patients with ‘partial WFS1 protein’ possessed *WFS1* missense variants that had differing results in pathogenicity on in-silico analysis. S07, who had an *OPA1* variant, was reclassified as autosomal DOA with sensorineural deafness and type 1 DM and was excluded from this group for genotype–phenotype analysis. The other two patients have the following mean ages of onset: DM 3.8 years (range 1.5–6.0 years); OA 11 years (8–14); DI 13 years (SO1 only); hearing loss 5 years (1.5–14); and urinary dysfunction at 14.5 years (14–15). The mean HbA1c was 72.1 mmol/mol (65.2–79.0), the mean hearing loss range was 65 dB (50–80) and the logMAR value for current visual acuity was 0.3 (0.2–0.4).

**Figure 3:**
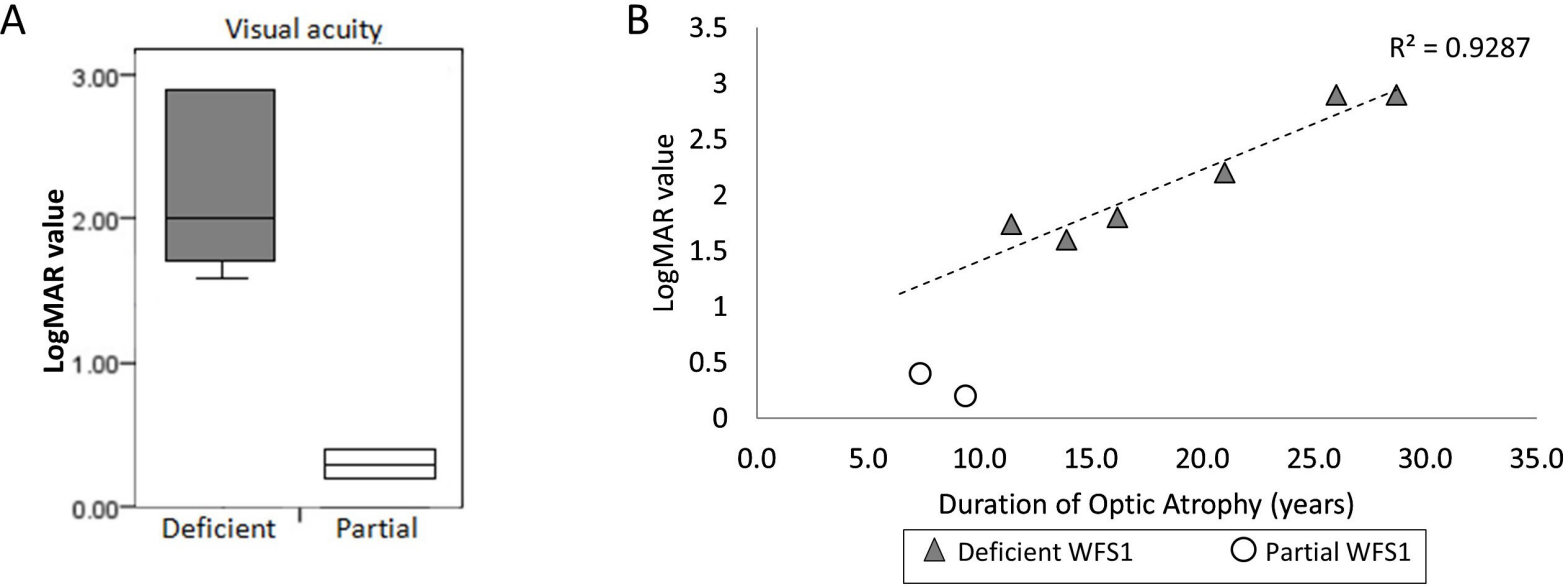
Comparison of visual acuity data between groups.**(A)** Box plot comparing LogMAR values between deficient (n=6) and partial WFS1 (n=2) protein groups; showing statistically significant difference (p=0.04). logMAR value (visual acuity logarithm of the minimum angle of resolution) is the magnification requirement, the higher the logMAR value, the worse the visual acuity (<1.0 mild-moderate visual impairment, 1.0-1.3 sight impaired (partial sighted) >1.3 severely sight impaired (blind). 40–42 **(B)** Scatter graph showing logMAR values of each patient corresponding to the duration of optic atrophy from the diagnosis of OA (irrespective of the age of diagnosis). Plots for patients in deficient WFS1 protein group shown in grey triangles (n=6); regression line is drawn between the patients in deficient WFS1 protein group (correlation of determination is 0.93). Plots for patients in partial WFS1 protein group (SO1 + SO2) shown in white circles (n=2).

Comparing the two groups, there was a statistically significant difference in the severity of OA as measured by current logMAR score (p=0.04) (figure 3A), but not in the age of onset of OA (p=0.13) (online supplemental figure 1). The partial WFS1 protein group had better visual acuity on current logMAR value, meaning milder symptoms of OA. The degree of visual impairment remained markedly different between the groups, even after taking the duration of OA (from diagnosis) into account (figure 3B).

10.1136/jmedgenet-2020-107257.supp1 Supplementary data

S01 has colour vision deficiency and up until the age of 19 years (2017) had visual acuity within the normal range but has recently been denied a driving licence. S02 has had long-standing asymptomatic OA since the first examination and can read normal size text. The vision of S07 has been stable, with incremental changes to refractory prescriptions and right temporal retinal fibre layer loss, characteristic of patients with OPA1 DOA. This differs from WFS, which tends to produce diffuse OA.

By comparison, in the ‘deficient WFS1 protein’ group, all are registered severely visually impaired: requiring the use of Braille, computer speech software or size 72 font and/or using guide dogs.

There were no qualitative differences in the MRI brain reports between the two groups. All the patients had radiological evidence of atrophy of optic nerve, chiasm and tracts, except patient S07 where MRI was unable to be undertaken.

There were no statistically significant differences in urinary dysfunction, mean levels of HbA1c or degree of hearing loss, nor in the age of onset of DM, OA, DI and hearing loss, between the groups (online supplemental figure 1) and (online supplemental table 1).

## Discussion

### Correlation of WFS1 protein level and severity of OA

Data from a patient-reported outcomes measures survey completed by 48 patients with WFS in the UK revealed that vision impairment was the most important symptom affecting the quality of life (Barrett TG 2018, unpublished).

We have shown that the six patients with a clinical diagnosis of WFS, who are compound heterozygous for loss of function variants, or a missense variant, and predicted to be damaging in multiple protein prediction software programmes, have no detectable WFS1 protein expression. These patients had the onset of OA at a median age of 5.5 years, and progression to severe vision impairment (logMAR >1.0) within 11.4 years of onset.

Two patients with the *WFS1* variants p.Glu169Lys and p.His313Tyr are associated with having residual WFS1 protein expression; their OA commenced at a median age of 11 years, and they have maintained only mildly or moderately reduced visual acuity (logMAR scores 0.5 and 0.2) despite having 8.4 years mean duration of OA (figure 3B). The presence of residual WFS1 protein expression in patients S01 and S02 appears to be associated with a later onset as well as the markedly slower progression of visual impairment.

Given the rarity of WFS, the fact that this correlation was seen in both of the patients who we discovered to have residual WFS1 levels, this will need to be investigated in other patients with residual WFS1 protein expression.

One patient (S07) was found to have *OPA1* mutation and the heterozygous p.Glu385Lys *WFS1* missense variant. Due to the possible confounding effect of the *OPA1* mutation, S07 was not included in the genotype–phenotype analysis for OA. However, S07 was included in this study because the patient initially presented clinically with characteristics of WFS, with milder symptoms of OA. Even though S07 does not have classical WFS based on the genotype, the fact that the fibroblasts only had 44.6% of WFS1 protein suggests that this *WFS1* variant may confer some pathogenicity. Also, we cannot exclude the possibility of an interaction between the *WFS1* and *OPA1* variants contributing to their OA. Therefore this patient was worthy of the further discussion below.

### WFS1 variants and WFS mRNA and protein levels

Our results show that irrespective of WFS1 protein levels, all the patients have 33%–47% of *WFS1* mRNA still present, including patients with nonsense mutations. This suggests that WFS1 mRNA from these variants may not have been destroyed by nonsense-mediated mRNA decay (NMD). This is consistent with reports of patients with WFS harbouring homozygous Trp700X variants and the Phe343fsX396 variant who did not trigger NMD.^28^ According to the rule that to trigger NMD, the premature stop codons must lie 50–55 nucleotides upstream from the last exon/exon junction^29^; only premature stop codons upstream of the amino acid position 269 in exon 8 of WFS1 should cause NMD. All nonsense variants in our study patients were downstream of this position.

The absence of any detectable truncated WFS1 proteins in the deficient WFS1 protein group, by immunoblotting, is likely a result of the instability of these abnormal proteins. This is consistent with a report that demonstrated no detectable WFS1 protein in a patient harbouring homozygous nonsense mutations and markedly reduced WFS1 protein in a patient harbouring missense and nonsense variants. Results from pulse-chase experiment suggest that these WFS1 mutations resulted in an unstable WFS1 protein with a markedly reduced half-life.^28^

### ER stress response

Of the patients with reduced WFS1 residual protein level (S02 and S07), we did not detect any consistent increase in any ER stress markers. Protein expression from the wild type WFS1 allele appears to offer sufficient protection against ER stress. This is consistent with the expected WFS1 protein expression in obligate *WFS1* variant carriers who do not express a WFS phenotype.

WFS1 protein is a negative regulator of the ATF6 transcription factor, and in WFS1 depletion, hyperactivation of the ATF6 pathway was described.^9^ We demonstrated activation of ATF6 pathway in fibroblasts of patients S02 and S10 in our luciferase reporter assay. In S10, this is likely due to depletion of WFS1 protein, which is consistent with the previous report.^9^ In S02, the activation of the ATF6 pathway likely resulted from a dominant-negative effect of the c.937C>T;p.His313Tyr allele. Functional assays have shown that mutant p.His313Tyr WFS protein significantly increases ER stress and shows autosomal dominant inheritance.^23 24^

The analysis of the other ER stress markers between the groups proved inconclusive. Fibroblasts are cells that are not known to harbour pathology in patients with WFS. Interestingly, impairment of calcium homeostasis was reported in *WFS1*-deficient fibroblasts from patients with WFS.^30^ We have previously demonstrated evidence of ER stress and impaired ER calcium homeostasis in more disease-relevant tissues.^31^

### WFS1 c.505G>A;p.Glu169Lys variant

Patient S01 has a compound heterozygous nonsense (c.1558C>T;p.Gln520X) and a previously reported missense (c.505G>A;p.Glu169Lys) WFS1 variant.^32^ This genotype resulted in a 96.2% reduction of WFS1 protein levels, suggesting that this missense mutation resulted in marked instability of the WFS1 protein.

S01 has classical features of WFS. After having initial isolated colour vision reduction, S01 progressed to using an iPad to photograph the whiteboard in classrooms. The patient was initially considered for a driving licence but was turned down due to the vision impairment.

The consequences of this genotype, resulting in 3.8% residual WFS1 protein, appear to correlate with a slower progression of visual impairment, compared with patients with a complete absence of WFS1 protein expression.

### WFS1 c.937C>T;p.His313Tyr variant

Patient S02 has a ‘de novo’ *WFS1* c.937C>T;p.His313Tyr variant as well as a duplication in an untranslated region of *WFS1*. This patient had profound early-onset hearing loss and DM, both by 18 months. OA was diagnosed at 8 years but remains asymptomatic. We have shown a 52.3% reduction in WFS1 protein levels in the fibroblasts (figure 1B). Our results from ER stress luciferase reporter transfected to fibroblasts from S02 also demonstrated increased ATF6-dependent UPR activation (figure 1D).

There have been two other reported cases of unrelated patients with WFS who were also found to have a single de novo His313Tyr *WFS1* variant, respectively.^6 23^ Interestingly, they also developed profound early hearing loss by 2 years of age, DM by 4 years of age and had learning impairment.^6^ His313Tyr was subsequently shown to be disease associated in an autosomal dominant manner in a cell model for WFS.^24^ The clinical features in all three of these patients with His313Tyr variant are typical of patients with autosomal dominant WFS, with a spectrum that includes neonatal/infancy onset DM, congenital cataracts and sensorineural deafness.^23^ Our patient has not yet shown any evidence of cataracts.

S02 also has a six base pair duplication (c.1709_14dupTGCCCC) in the 5′ untranslated region of exon 1. This was inherited from the patient’s mother, who is an asymptomatic carrier. The duplication is located within the WFS1 minimal promoter region and a critical GC box. Deletions in this area affect transcription factor binding and reduce gene transcription.^33^ However, the effect of this particular duplication on WFS1 promoter activity is unknown. Therefore, we are unable to determine to what degree this six base pair duplication or the p.His313Tyr variant had on the 52.3% reduction in WFS1 protein levels we observed.

### WFS1 c.1153G>A;p.Glu385Lys variant

Patient S07 has a heterozygous *WFS1* missense variant (c.1153G>A;p.Glu385Lys) and a heterozygous *OPA1* duplication variant (duplication of exons 4–8) found following further genetic screening. Similar duplications within *OPA1* have been reported as disease associated.^34–36^

S07 had initial clinical features fulfilling clinical criteria for WFS including DM, OA and hearing loss.

This patient was born with profound sensorineural hearing loss due to non-functioning cochlear, requiring bilateral cochlear implants. Weakly positive anti-GAD65 antibodies suggested a possible autoimmune cause of DM. OA was first recognised at 4 years. S07 has had a slow progression of OA, requiring only incremental changes to prescription glasses. This patient’s current corrected visual acuity is borderline for a private vehicle driving licence.

After the family genetic screening, it was found that this patient inherited both the *OPA1* and *WFS1* variants from their mother and maternal grandfather (figure 1A).^5^ The patient’s mother had type 1 DM since 16 years of age. Subsequently, after positive *OPA1* screening, bilateral temporal OA was seen at 39 years of age, with logMAR 0.2 acuity in each eye. Maternal hearing is normal, and she is otherwise well. Maternal grandfather has isolated bilateral OA but no diabetes.

We conclude that the phenotype of S07 may best be explained as autosomal DOA due to the *OPA1* variant, with associated hearing loss (DOA-plus syndrome, seen in 20% of cases of DOA).^37^ S07 has temporal OA and nerve fibre layer loss in a characteristic pattern for *OPA1* DOA.^38^ Interestingly, this particular *OPA1* variant has not been previously reported to result in a DOA-plus phenotype.^39^ Their autoimmune type 1 DM is assumed to be coincidental.

The c.1153G>A;p.Glu385Lys *WFS1* missense variant that we detected has not been previously reported in patients with WFS and is currently of unknown significance. However, it has been reported in patients without WFS, who had either isolated OA or sensorineural deafness.^25–27^ It remains a possibility that there may be an interaction between this *WFS1* variant and the *OPA1* duplication that could have contributed to S07’s development of sensorineural deafness and OA.

We have also shown there was a 55.4% reduction in WFS1 protein levels observed in S07’s fibroblasts. Therefore, we speculate that this *WFS1* variant may result in classical WFS if in combination with another loss of function *WFS1* variant.

### Conclusion

We have shown that residual WFS1 protein levels in patients with WFS show milder visual impairment and slower progression compared with patients with absent protein expression. Even a minimal WFS1 protein expression of 3.8% of wild type seems to have an ameliorating effect.

Our findings suggest that there may be a therapeutic benefit in strategies to increase residual WFS1 protein levels for those patients who retain some protein expression.

## Data Availability

All data relevant to the study are included in the article or uploaded as supplementary information. All data relevant to the study are included in the article.

## References

[1] Barrett TG , Bundey SE , Macleod AF , Neurodegeneration MAF . Neurodegeneration and diabetes: UK nationwide study of Wolfram (DIDMOAD) syndrome. Lancet 1995;346:1458–63. 10.1016/S0140-6736(95)92473-6 7490992

[2] Urano F . Wolfram syndrome: diagnosis, management, and treatment. Curr Diab Rep 2016;16:6. 10.1007/s11892-015-0702-6 26742931 PMC4705145

[3] Inoue H , Tanizawa Y , Wasson J , Behn P , Kalidas K , Bernal-Mizrachi E , Mueckler M , Marshall H , Donis-Keller H , Crock P , Rogers D , Mikuni M , Kumashiro H , Higashi K , Sobue G , Oka Y , Permutt MA . A gene encoding a transmembrane protein is mutated in patients with diabetes mellitus and optic atrophy (Wolfram syndrome). Nat Genet 1998;20:143–8. 10.1038/2441 9771706

[4] Tranebjaerg L , Barrett T , Rendtorff ND . WFS1-Related Disorders. GeneReviews((R)). Seattle (WA): University of Washington, Seattle University of Washington, Seattle. GeneReviews is a registered trademark of the University of Washington, Seattle. All rights reserved 1993.

[5] Astuti D , Sabir A , Fulton P , Zatyka M , Williams D , Hardy C , Milan G , Favaretto F , Yu-Wai-Man P , Rohayem J , López de Heredia M , Hershey T , Tranebjaerg L , Chen J-H , Chaussenot A , Nunes V , Marshall B , McAfferty S , Tillmann V , Maffei P , Paquis-Flucklinger V , Geberhiwot T , Mlynarski W , Parkinson K , Picard V , Bueno GE , Dias R , Arnold A , Richens C , Paisey R , Urano F , Semple R , Sinnott R , Barrett TG . Monogenic diabetes syndromes: locus-specific databases for Alström, Wolfram, and thiamine-responsive megaloblastic anemia. Hum Mutat 2017;38:764–77. 10.1002/humu.23233 28432734 PMC5535005

[6] Hansen L , Eiberg H , Barrett T , Bek T , Kjaersgaard P , Tranebjaerg L , Rosenberg T . Mutation analysis of the WFS1 gene in seven Danish Wolfram syndrome families; four new mutations identified. Eur J Hum Genet 2005;13:1275–84. 10.1038/sj.ejhg.5201491 16151413

[7] Fonseca SG , Fukuma M , Lipson KL , Nguyen LX , Allen JR , Oka Y , Urano F . Wfs1 is a novel component of the unfolded protein response and maintains homeostasis of the endoplasmic reticulum in pancreatic beta-cells. J Biol Chem 2005;280:39609–15. 10.1074/jbc.M507426200 16195229

[8] Ariyasu D , Yoshida H , Hasegawa Y , Reticulum E . Er) stress and endocrine disorders. Int J Mol Sci 2017;18:382. 10.3390/ijms18020382 PMC5343917 28208663

[9] Fonseca SG , Ishigaki S , Oslowski CM , Lu S , Lipson KL , Ghosh R , Hayashi E , Ishihara H , Oka Y , Permutt MA , Urano F . Wolfram syndrome 1 gene negatively regulates ER stress signaling in rodent and human cells. J Clin Invest 2010;120:744–55. 10.1172/JCI39678 20160352 PMC2827948

[10] Rohayem J , Ehlers C , Wiedemann B , Holl R , Oexle K , Kordonouri O , Salzano G , Meissner T , Burger W , Schober E , Huebner A , Lee-Kirsch MA . Wolfram syndrome diabetes writing G. diabetes and neurodegeneration in Wolfram syndrome: a multicenter study of phenotype and genotype. Diabetes Care 2011;34:1503–10. 21602428 10.2337/dc10-1937 PMC3120194

[11] Matsunaga K , Tanabe K , Inoue H , Okuya S , Ohta Y , Akiyama M , Taguchi A , Kora Y , Okayama N , Yamada Y , Wada Y , Amemiya S , Sugihara S , Nakao Y , Oka Y , Tanizawa Y . Wolfram syndrome in the Japanese population; molecular analysis of WFS1 gene and characterization of clinical features. PLoS One 2014;9:e106906. 10.1371/journal.pone.0106906 25211237 PMC4161373

[12] Grenier J , Meunier I , Daien V , Baudoin C , Halloy F , Bocquet B , Blanchet C , Delettre C , Esmenjaud E , Roubertie A , Lenaers G , Hamel CP . Wfs1 in optic neuropathies: mutation findings in nonsyndromic optic atrophy and assessment of clinical severity. Ophthalmology 2016;123:1989–98. 10.1016/j.ophtha.2016.05.036 27395765

[13] Elli FM , Ghirardello S , Giavoli C , Gangi S , Dioni L , Crippa M , Finelli P , Bergamaschi S , Mosca F , Spada A , Beck-Peccoz P . A new structural rearrangement associated to Wolfram syndrome in a child with a partial phenotype. Gene 2012;509:168–72. 10.1016/j.gene.2012.06.077 22771918

[14] Farmer A , Aymé S , de Heredia ML , Maffei P , McCafferty S , Młynarski W , Nunes V , Parkinson K , Paquis-Flucklinger V , Rohayem J , Sinnott R , Tillmann V , Tranebjaerg L , Barrett TG . EURO-WABB: an EU rare diseases Registry for Wolfram syndrome, Alström syndrome and Bardet-Biedl syndrome. BMC Pediatr 2013;13:130. 10.1186/1471-2431-13-130 23981649 PMC3765797

[15] van Beeck Calkoen EA , Engel MSD , van de Kamp JM , Yntema HG , Goverts ST , Mulder MF , Merkus P , Hensen EF . The etiological evaluation of sensorineural hearing loss in children. Eur J Pediatr 2019;178:1195–205. 10.1007/s00431-019-03379-8 31152317 PMC6647487

[16] Ng PC , Henikoff S . SIFT: predicting amino acid changes that affect protein function. Nucleic Acids Res 2003;31:3812–4. 10.1093/nar/gkg509 12824425 PMC168916

[17] Adzhubei I , Jordan DM , Sunyaev SR . *Curr Protoc Hum Genet* 2013;Chapter 7:Unit7 20. In: Predicting functional effect of human missense mutations using PolyPhen-2. (published Online First: 2013/01/15). 10.1002/0471142905.hg0720s76 PMC4480630 23315928

[18] Schwarz JM , Rödelsperger C , Schuelke M , Seelow D . MutationTaster evaluates disease-causing potential of sequence alterations. Nat Methods 2010;7:575–6. 10.1038/nmeth0810-575 20676075

[19] Choi Y , Sims GE , Murphy S , Miller JR , Chan AP . Predicting the functional effect of amino acid substitutions and indels. PLoS One 2012;7:e46688–e88. 10.1371/journal.pone.0046688 23056405 PMC3466303

[20] Kokame K , Kato H , Miyata T . Identification of ERSE-II, a new cis-acting element responsible for the ATF6-dependent mammalian unfolded protein response. J Biol Chem 2001;276:9199–205. 10.1074/jbc.M010486200 11112790

[21] Hardy C , Khanim F , Torres R , Scott-Brown M , Seller A , Poulton J , Collier D , Kirk J , Polymeropoulos M , Latif F , Barrett T . Clinical and molecular genetic analysis of 19 Wolfram syndrome kindreds demonstrating a wide spectrum of mutations in WFS1. Am J Hum Genet 1999;65:1279–90. 10.1086/302609 10521293 PMC1288280

[22] Kwak SH , Jung C-H , Ahn CH , Park J , Chae J , Jung HS , Cho YM , Lee DH , Kim J-I , Park KS . Clinical whole exome sequencing in early onset diabetes patients. Diabetes Res Clin Pract 2016;122:71–7. 10.1016/j.diabres.2016.10.005 27810688

[23] De Franco E , Flanagan SE , Yagi T , Abreu D , Mahadevan J , Johnson MB , Jones G , Acosta F , Mulaudzi M , Lek N , Oh V , Petz O , Caswell R , Ellard S , Urano F , Hattersley AT . Dominant ER Stress-Inducing WFS1 Mutations Underlie a Genetic Syndrome of Neonatal/Infancy-Onset Diabetes, Congenital Sensorineural Deafness, and Congenital Cataracts. Diabetes 2017;66:2044–53. 10.2337/db16-1296 28468959 PMC5482085

[24] Bonnycastle LL , Chines PS , Hara T , Huyghe JR , Swift AJ , Heikinheimo P , Mahadevan J , Peltonen S , Huopio H , Nuutila P , Narisu N , Goldfeder RL , Stitzel ML , Lu S , Boehnke M , Urano F , Collins FS , Laakso M . Autosomal dominant diabetes arising from a Wolfram syndrome 1 mutation. Diabetes 2013;62:3943–50. 10.2337/db13-0571 23903355 PMC3806620

[25] Cryns K , Sivakumaran TA , Van den Ouweland JMW , Pennings RJE , Cremers CWRJ , Flothmann K , Young T-L , Smith RJH , Lesperance MM , Van Camp G . Mutational spectrum of the WFS1 gene in Wolfram syndrome, nonsyndromic hearing impairment, diabetes mellitus, and psychiatric disease. Hum Mutat 2003;22:275–87. 10.1002/humu.10258 12955714

[26] Kytövuori L , Seppänen A , Martikainen MH , Moilanen JS , Kamppari S , Särkioja T , Remes AM , Räsänen P , Rönnemaa T , Majamaa K . Wfs1 variants in Finnish patients with diabetes mellitus, sensorineural hearing impairment or optic atrophy, and in suicide victims. J Hum Genet 2013;58:495–500 https://www.nature.com/articles/jhg201329#supplementary-information 10.1038/jhg.2013.29 23595122

[27] Häkli S , Kytövuori L , Luotonen M , Sorri M , Majamaa K . Wfs1 mutations in hearing-impaired children. Int J Audiol 2014;53:446–51. 10.3109/14992027.2014.887230 24909696

[28] Hofmann S , Bauer MF . Wolfram syndrome-associated mutations lead to instability and proteasomal degradation of Wolframin. FEBS Lett 2006;580:4000–4. 10.1016/j.febslet.2006.06.036 16806192

[29] Nagy E , Maquat LE . A rule for termination-codon position within intron-containing genes: when nonsense affects RNA abundance. Trends Biochem Sci 1998;23:198–9. 10.1016/S0968-0004(98)01208-0 9644970

[30] Angebault C , Fauconnier J , Patergnani S , Rieusset J , Danese A , Affortit CA , Jagodzinska J , Mégy C , Quiles M , Cazevieille C , Korchagina J , Bonnet-Wersinger D , Milea D , Hamel C , Pinton P , Thiry M , Lacampagne A , Delprat B , Delettre C . ER-mitochondria cross-talk is regulated by the Ca^2+^ sensor NCS1 and is impaired in Wolfram syndrome. Sci Signal 2018;11. 10.1126/scisignal.aaq1380. [Epub ahead of print: 23 OCt 2018]. 30352948

[31] Zatyka M , Da Silva Xavier G , Bellomo EA , Leadbeater W , Astuti D , Smith J , Michelangeli F , Rutter GA , Barrett TG , Sarco BTG . Sarco(endo)plasmic reticulum ATPase is a molecular partner of Wolfram syndrome 1 protein, which negatively regulates its expression. Hum Mol Genet 2015;24:814–27. 10.1093/hmg/ddu499 25274773 PMC4291252

[32] Khanim F , Kirk J , Latif F , Barrett TG . Wfs1/Wolframin mutations, Wolfram syndrome, and associated diseases. Hum Mutat 2001;17:357–67. 10.1002/humu.1110 11317350

[33] Ricketts C , Zatyka M , Barrett T . The characterisation of the human Wolfram syndrome gene promoter demonstrating regulation by Sp1 and Sp3 transcription factors. Biochim Biophys Acta 2006;1759:367–77. 10.1016/j.bbaexp.2006.06.005 16965966

[34] Fuhrmann N , Alavi MV , Bitoun P , Woernle S , Auburger G , Leo-Kottler B , Yu-Wai-Man P , Chinnery P , Wissinger B . Genomic rearrangements in OPA1 are frequent in patients with autosomal dominant optic atrophy. J Med Genet 2009;46:136–44. 10.1136/jmg.2008.062570 19181907

[35] Fuhrmann N , Schimpf S , Kamenisch Y , Leo-Kottler B , Alexander C , Auburger G , Zrenner E , Wissinger B , Alavi MV . Solving a 50 year mystery of a missing OPA1 mutation: more insights from the first family diagnosed with autosomal dominant optic atrophy. Mol Neurodegener 2010;5:25. 10.1186/1750-1326-5-25 20546606 PMC2893178

[36] Eiberg H , Hansen L , Kjer B , Hansen T , Pedersen O , Bille M , Rosenberg T , Tranebjaerg L . Autosomal dominant optic atrophy associated with hearing impairment and impaired glucose regulation caused by a missense mutation in the WFS1 gene. J Med Genet 2006;43:435–40. 10.1136/jmg.2005.034892 16648378 PMC2649014

[37] Yu-Wai-Man P , Griffiths PG , Gorman GS , Lourenco CM , Wright AF , Auer-Grumbach M , Toscano A , Musumeci O , Valentino ML , Caporali L , Lamperti C , Tallaksen CM , Duffey P , Miller J , Whittaker RG , Baker MR , Jackson MJ , Clarke MP , Dhillon B , Czermin B , Stewart JD , Hudson G , Reynier P , Bonneau D , Marques W , Jr. LG , McFarland R , Taylor RW , Turnbull DM , Votruba M , Zeviani M , Carelli V , Bindoff LA , Horvath R . Amati-Bonneau P, Chinnery PF. multi-system neurological disease is common in patients with OPA1 mutations. Brain 2010;133:771–86. 20157015 10.1093/brain/awq007 PMC2842512

[38] Lenaers G , Hamel C , Delettre C , Amati-Bonneau P , Procaccio V , Bonneau D , Reynier P , Milea D . Dominant optic atrophy. Orphanet J Rare Dis 2012;7:46. 10.1186/1750-1172-7-46 22776096 PMC3526509

[39] Ferré M , Bonneau D , Milea D , Chevrollier A , Verny C , Dollfus H , Ayuso C , Defoort S , Vignal C , Zanlonghi X , Charlin J-F , Kaplan J , Odent S , Hamel CP , Procaccio V , Reynier P , Amati-Bonneau P . Molecular screening of 980 cases of suspected hereditary optic neuropathy with a report on 77 novel OPA1 mutations. Hum Mutat 2009;30:E692–705. 10.1002/humu.21025 19319978

[40] WHO . International Statistical Classification of Diseases and Related Health Problems. 10th Revision. Chapter VII. H54. Visual impairment including blindness (binocular or monocular): World Health Organisation; 1990 [cited 2019 22nd May]. Version for 2016. Available: https://icd.who.int/browse10/2016/en#/H54.0

[41] Tadić V , Cumberland PM , Lewando-Hundt G , Rahi JS , vision-related quality of life group . Do visually impaired children and their parents agree on the child's vision-related quality of life and functional vision? Br J Ophthalmol 2017;101:244–50. 10.1136/bjophthalmol-2016-308582 27267607 PMC5339549

[42] Colenbrander A . Aspects of vision loss – visual functions and functional vision. Vis Impair Res 2003;5:115–36. 10.1080/1388235039048919

