# Supplementary material for: WFS1 protein expression correlates with clinical progression of optic atrophy in patients with Wolfram syndrome
Source: J Med Genet. 2021 May 18;59(1):65–74. doi: 10.1136/jmedgenet-2020-107257 (PMC8685651; doi:10.1136/jmedgenet-2020-107257)
Supplement: Supplementary data [file jmedgenet-2020-107257supp001.pdf]

APPENDIX: SUPPLEMENTARY TABLE AND FIGURE

SUPPLEMENTARY TABLE: Median with upper and lower values of baseline characteristics between partial and deficient WFS1 group. Data expressed in graphical form in Figure 3 and Supplementary Figure.

	WFS expression group		
	Partial (n=2)	*Deficient (n=6)*	*Significance*
Median (lower, upper) onset of DM (years)	*3.8 (1.5, 6.0)*	*5.5 (3.0, 10.0)*	*0.50*
Median (lower, upper) HbA1C (mmol/mol)	*72.1 (65.2, 79.0)*	*63.4 (55.2, 79.8)*	*0.32*
Median onset of OA (years)	*11.0 (8.0, 14.0)*	*5.5 (4.0, 9.0)*	*0.13*
Median current LogMAR value	*0.3 (0.2, 0.4)*	*2.0 (1.6, 2.9)*	*0.04*
Median onset of hearing loss (years)	*7.8 (1.5, 14.0)*	*8.5 (4.0, 13.0)*	*1.00*
Median hearing threshold (dB)	*65.0 (50, 80)*	*77.5 (35.0, 100.0)*	*0.64*
Median onset of urinary dysfunction (years)	*13.0 (13.0)*	*10.5 (3.0, 16.0)*	*1.00*
Median onset of DI (years)	*14.5 (14.0, 15.0)*	*16.0 (10.0, 16.0)*	*0.33*

SUPPLEMENTARY FIGURE: Relation of phenotype with WFS1 protein expression

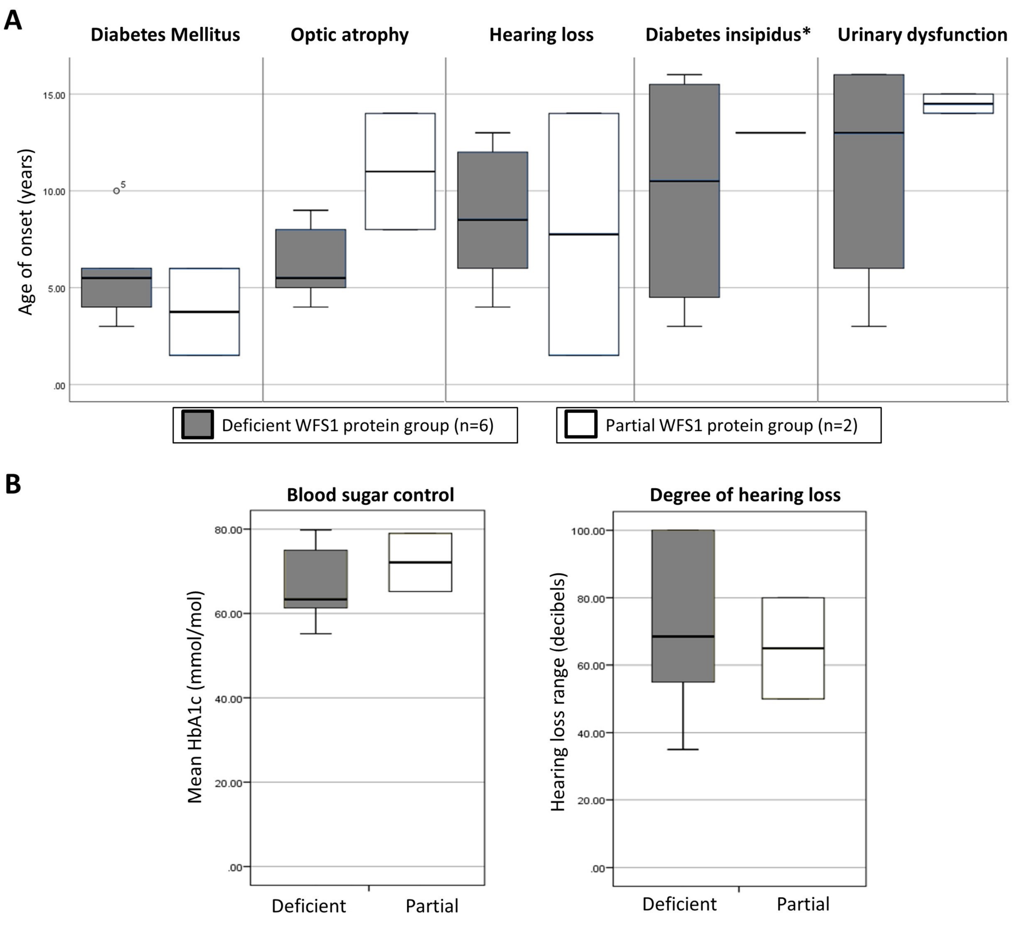

(A) Box plots comparing the age of onset for Diabetes mellitus, optic atrophy, hearing loss, diabetes insipidus and urinary dysfunction, between deficient and partial WFS1 protein groups. p>0.05 between deficient and partial WFS1 protein groups. * 2 patients in deficient group not developed DI, 1 patient in the partial WFS1 group not developed DI.

(B) Box plot comparing HbA1c and hearing loss threshold between deficient and partial WFS1 protein groups. P>0.05.
